# 617. Long Acting Lipoglycopeptide Use in Veterans for Serious Gram-Positive Infections in the COVID Era

**DOI:** 10.1093/ofid/ofab466.815

**Published:** 2021-12-04

**Authors:** Carlos S Saldana, Tiffany Goolsby, Lauren H Epstein, Nora Oliver

**Affiliations:** 1 Emory University School of Medicine, Atlanta, Georgia; 2 Atlanta VA Health Care System, Atlanta, Georgia; 3 Veterans Administration Hospital, Decatur, Georgia; 4 Emory University School of Medicine, Atlanta VAMC, and Georgia Emerging Infections Program, Atlanta, Georgia

## Abstract

**Background:**

Dalbavancin and Oritavancin are semisynthetic lipoglycopeptides (LGP) that are FDA-approved for treatment of skin and soft tissue infections, but emerging data supports LGP use for other serious gram positive (GP) infections. We describe our experience with LGP during the COVID-19 pandemic.

**Methods:**

We initiated a quality improvement project to assess the use of LGP for label and off-label indications at the Atlanta Veterans Affairs Health Care System. We define serious GP infections as infective endocarditis, osteomyelitis, joint infections, or bacteremia. Patients with serious GP infections that receivedLGP were selected at the treating physician's discretion. We reviewed medical records of all patients receiving at least one dose of long-acting LGP from March 1, 2020 - May 31, 2021. We described patient demographics, clinical information,and outcomes (90-day readmission).

**Results:**

Nineteen patients with GP infections received LGP (table). Overall, the most common infection was cellulitis 7 (35%); 14 patients received LGPs for serious GP infections. All patients received at least one other non-LGP antibiotic for at least 2 days, majority vancomycin (60%) and cefazolin (30%). Overall, the median hospital stay among patients who received LGP was 8.5 days (range: 2-45 days), for those with serious GP infections the median hospital stay was 15 days (range: 4-45). 90% of patientswho received LGP were discharged home. Number of LGP doses ranged from 1 to 6 doses total, based on type of infection. Sixteen veterans (80%) followed up in outpatient clinicfollowing discharge within 2 weeks, two patients were discharged to home hospice due to complications of underlying malignancies and two patients were lost to follow up. Noadverse drug events were reported, and none with serious GP infections required rehospitalization at 90 days.

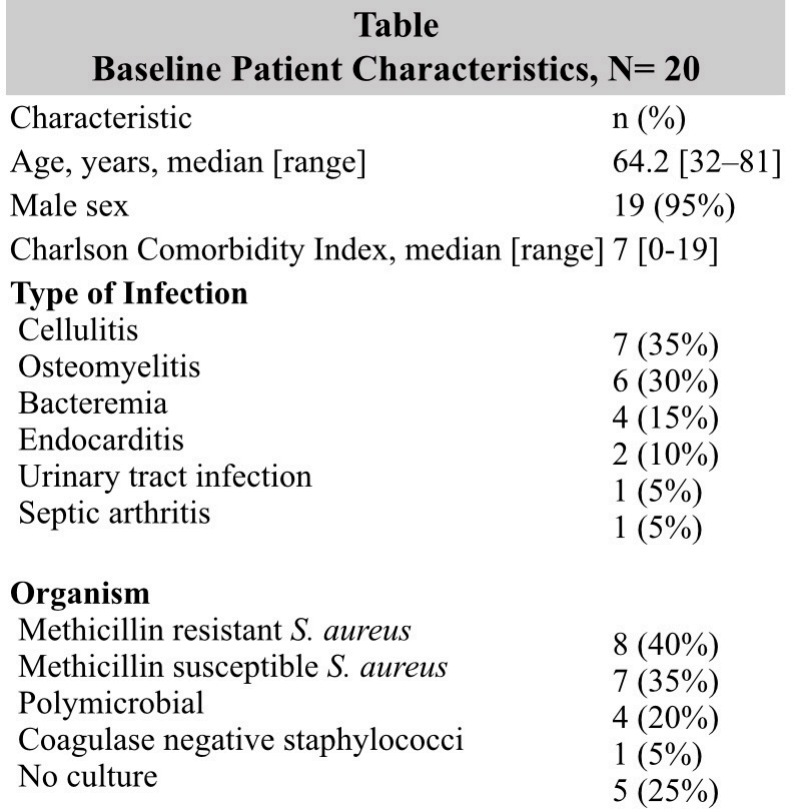

**Conclusion:**

Our experience suggests that long-acting LGP may be valuable tools to treat serious gram-positive infections by optimizing theduration of hospitalization and preventing unnecessary admissions to acute care and nursing facilities for daily antibiotic infusions. These aspects of LGP use are especially important during the COVID-19 pandemic where nosocomial transmission has been documented.

**Disclosures:**

**All Authors**: No reported disclosures

